# An Evidence-Tiered Biomimetic Design Space Workflow for the Conceptual Design of Elderly-Care Robots

**DOI:** 10.3390/biomimetics11080578

**Published:** 2026-08-13

**Authors:** Wangshuang Zang, Congrong Xiao, Dongkwon Seong

**Affiliations:** 1 School of Design and Innovation, Guangzhou University of Software, Guangzhou 510990, China; zws@mail.gzus.edu.cn; 2 College of Art and Design, Guangdong Baiyun University, Guangzhou 510900, China; xiaocongrong@baiyunu.edu.cn; 3 Department of X-Cultural Studies, Kookmin University Graduate School, Kookmin University, Seoul 02707, Republic of Korea

**Keywords:** biomimetic design, elderly-care robot, evidence-tiered translation, constrained design space exploration, Latin hypercube sampling, braking distance proxy, conceptual CAD, AI-assisted visualization

## Abstract

Background: Early-stage elderly-care robot design requires biological analogies to be translated without turning qualitative inspiration into unvalidated numerical evidence. Methods: We audited 15 initial variables and retained a four-dimensional exploratory space: nominal shell-edge radius (V06), pre-braking trigger distance (V09), translational speed (V11), and commanded deceleration (V12). Latin hypercube samples were filtered by V09 − V11^2^/(2V12) ≥ 0. A derived warning margin proxy, I5 = 1 − [V11^2^/(2V12)]/V09, was evaluated with fixed-seed feasibility, distribution, coverage, cluster, coordinate stability, and distance-sensitivity diagnostics. Results: The pooled pre-check acceptance rate was 0.8621. I5 descriptive and distributional stability passed at *N* = 768→1024, but four-dimensional coverage passed in only 10/30 seeds; no sufficient *N* was established up to 1024. Natural cluster structure was not detected, exact representative coordinates were seed-sensitive, and selection overlap under an alternative distance definition was 0.53. Two fixed-seed points were therefore retained only as illustrative boundaries. AI-assisted images and legacy Rhino studies were used for qualitative design communication, not as validated realizations of the computation. Conclusions: The evidence-tiered workflow supports traceable exclusion of infeasible combinations and transparent product design translation while preserving explicit limits: no physical safety, usability, manufacturing, or optimality claim is made.

## 1. Introduction

Population aging is increasing the demand for assistive technologies that support independent living, reduce caregiver burden, and maintain quality of life [1,2,3]. Mobile and socially assistive robots are consequently being investigated for monitoring, communication, reminders, logistics, and companionship. In domestic settings, however, an early milestone is the reconciliation of physical presence, approach behavior, legible feedback, and service logic.

Biomimetics can help organize these decisions by abstracting functions from biological systems and translating them into engineering roles [4,5,6,7,8,9,10,11]. The central integrity risk is overtranslation: a biological analogy may justify a design direction without supplying a validated numerical threshold, causal performance claim, or safety certificate. This risk is especially important for older users, for whom perceived safety, usability, trust, and acceptance require direct human-centered evidence [12,13,14,15].

The initial study combined 15 variables, objective weighting, and alternative ranking. A revision audit found that several variables were undefined, duplicated another construct, or lacked an evidenced link to the score. Retaining those elements would create false precision. The present revision therefore treats the initial manuscript as the provenance baseline but replaces the invalidated ranking with a constrained, four-dimensional design space exploration.

This study aims to establish a traceable route from delimited biomimetic evidence to early-stage elderly-care robot concepts. It asks which candidate variables can be computationally operationalized, whether the constrained sample is sufficiently stable for reporting, and how computational outputs can inform product design without being presented as validated robot performance.

The contributions are: (1) an evidence-tiered mapping that distinguishes direct functional analogs, qualitative analogies, and ordinary engineering constraints; (2) a pre-specified, fixed-seed Latin hypercube exploration with explicit feasibility and stability diagnostics; and (3) an explicit handoff from computational coordinates to downstream morphology, interface, CMF, and conceptual CAD decisions.

The remainder of the article separates the evidence review, computational method, diagnostic results, product design translation, and validation needs so that each claim can be traced to its supporting evidence layer.

## 2. Literature Review

### 2.1. Biomimetic Design and Functional Abstraction

Established biomimetic processes move from problem definition and biological search to abstraction, transfer, and evaluation [4,5,6,7,8,9,10,11]. At the product level, the difficult step is not identifying an appealing organism but defining the smallest engineering role that the evidence supports. ISO 18458 supplies terminology and methodological context, but it does not validate project-specific robot dimensions or performance limits [11].

Biologically inspired design is used here as an umbrella term. Bionics commonly emphasizes the technical emulation of biological functions, whereas biomimetics focuses on the abstraction and transfer of biological principles into engineering applications. Biomimicry often extends this translation toward ecological-system analogies and broader sustainability objectives. Consistent with the terminology and methodological scope of ISO 18458 [11], the present study adopts a biomimetic framing because its central task is the delimited abstraction of biological functions and their product-level engineering translation. It does not claim literal organism imitation, ecosystem-level emulation, or sustainability performance.

### 2.2. Evidence for Product-Level Mechanism Translation

Two evidence relationships are sufficiently delimited for direct functional translation in this study. Turtle and tortoise carapaces support the abstraction of a curved protective envelope [8,16], while rodent vibrissae support a pre-contact sensing analogy [17]. Their engineering translations remain narrower than the biology: V06 is only a nominal shell-edge morphology coordinate, and V09 is only a longitudinal trigger distance construct.

Legible, non-threatening interface behavior and monitor–state–respond service logic are retained at qualitative tiers. Human–robot interaction studies support the relevance of sociability, legibility, and trust [13,18,19,20,21], while homeostasis offers a system-level analogy for adaptive regulation [22]. Neither tier supplies a sampled biological metric in the present computation.

### 2.3. Computational Design Space Exploration

Parametric exploration and multi-criteria methods can support early design decisions [23,24,25,26,27,28,29,30]. However, objective weighting and ranking do not justify the criteria themselves. If a criterion is unsupported or disconnected from the calculation, a precise weight or rank can conceal rather than resolve the evidential problem.

Latin hypercube sampling supports space-filling exploration of bounded variables [31,32]. For constrained spaces, reporting also requires checks on feasibility, distributional stability, coverage, cluster tendency, and sensitivity to the selection rule [33,34]. These diagnostics are particularly important when representative points are used downstream as design examples.

### 2.4. Research Gap and Study Positioning

Prior elderly-care robot research strongly supports the need for user evaluation [12,13,14,15], while biomimetic method research supports functional abstraction [4,5,6,7,8,9,10,11]. A gap remains between those studies: early product design studies need a transparent intermediate workflow that can explore a bounded concept space yet refuse unsupported claims before prototypes and user studies exist.

The present workflow addresses that gap through evidence tiers and explicit claim boundaries. Only M1 and M2 contribute computational variables. M3 and M4 guide qualitative design translation, and stability, ergonomics, emergency stop placement, material selection, and manufacturing remain ordinary downstream engineering tasks.

The candidate biological sources were inherited from the initial concept-development records and audited against the cited literature; the source set is not presented as a systematic or exhaustive biological search. This scope prevents a targeted concept study from implying database completeness that was not documented.

The study does not claim validated collision safety, elderly-user comfort, manufacturing feasibility, or statistical optimality. These outcomes require physical prototyping, measured system latency and stopping behavior, and human-centered evaluation.

## 3. Materials and Methods

### 3.1. Research Framework

**Figure 1:**
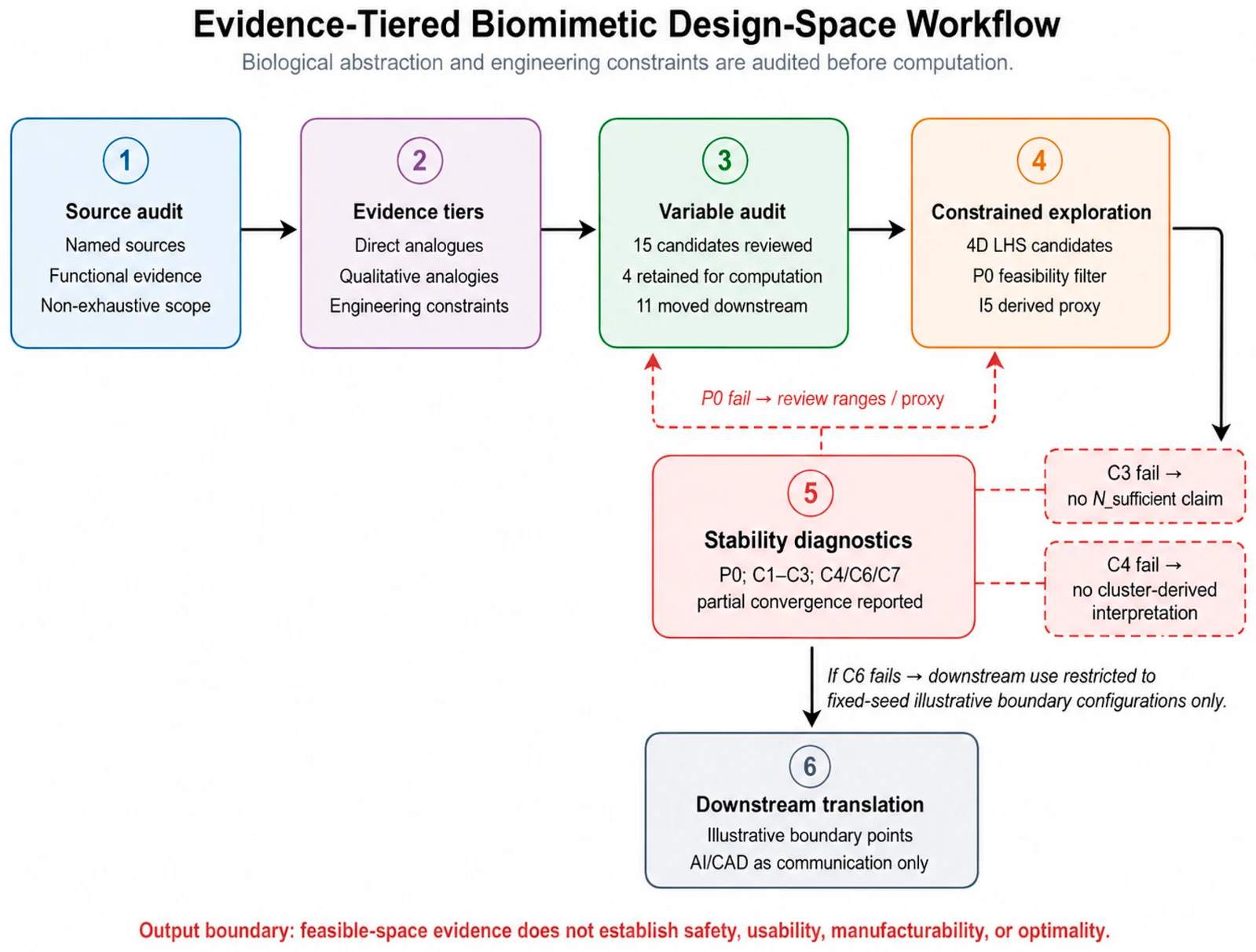
Evidence-tiered, constraint-based conceptual design workflow. The eight procedural steps are organized into six auditable modules. The main sequence progresses from source auditing and evidence classification to variable screening, constrained exploration, diagnostic evaluation, and claim-bounded downstream translation. Pre-specified return paths prevent failed feasibility, coverage, cluster tendency, or coordinate stability diagnostics from being interpreted as validated robot performance. (Author’s own work).

The revised workflow integrates evidence-tiered biomimetic abstraction, variable-scope auditing, constrained computational exploration, stability diagnostics, and downstream concept translation. It comprises eight procedural steps organized into six auditable modules. The workflow is sequential but not strictly linear: pre-specified return paths prevent failed feasibility, coverage, cluster tendency, or coordinate stability diagnostics from being converted into unsupported product claims (Figure 1).

(1) Selection and audit of named biological source systems and abstraction of delimited functional principles;
(2) Classification of direct functional analogs, qualitative analogies, and ordinary downstream engineering constraints;
(3) Audit of the 15 candidate variables for definition, evidence, independence, and computational influence;
(4) Definition of the final four-dimensional exploratory design space;
(5) Constrained Latin hypercube generation and rejection of combinations that fail the B9 feasibility condition;
(6) P0 feasibility checking and adaptive sample size diagnostics across fixed seeds;
(7) Assessment of coverage, cluster tendency, representative coordinate stability, and distance definition sensitivity; and
(8) Claim-bounded downstream translation into morphology, interface, CMF, AI-assisted visualization, and conceptual CAD.

The first four steps establish the admissible evidence and computational scope, Steps 5–7 evaluate whether the constrained exploration supports stable reporting, and Step 8 translates only those findings that remain within the established claim boundary. A failed P0 gate returns the workflow to range and proxy review. Failure of C3 prevents a sample size sufficiency claim. Failure of the C4 gate suppresses cluster-derived interpretation without terminating the remaining diagnostics. Failure of C6 restricts selected coordinates to fixed-seed illustrative boundary configurations. These return rules separate feasibility, distributional stability, coverage, and representative coordinate stability rather than treating them as interchangeable forms of validation.

The workflow does not use Entropy–CRITIC weighting, TOPSIS ranking, or an aggregate optimization score. This change was made because the audited criteria did not support a defensible single preference structure. The computational model instead characterizes a constrained feasible domain and reports whether the sampled distributions, coverage, and selected coordinates are stable under the pre-specified protocol.

### 3.2. Biomimetic Source Selection and Evidence-Tiered Framework

The candidate source set was inherited from the original concept-development records and audited against the cited peer-reviewed literature. This was a targeted source audit, not a systematic or exhaustive biological database search. Sources were retained only when the functional analog and product translation could be stated without exceeding the available evidence. Camouflage and mimicry were excluded because the study was delimited to near-contact domestic interaction and no clearly delimited engineering role was defined for them. M1 and M2 are direct functional analogs operationalized at restricted levels; M3 and M4 are qualitative interaction and system analogies.

**Figure 2:**
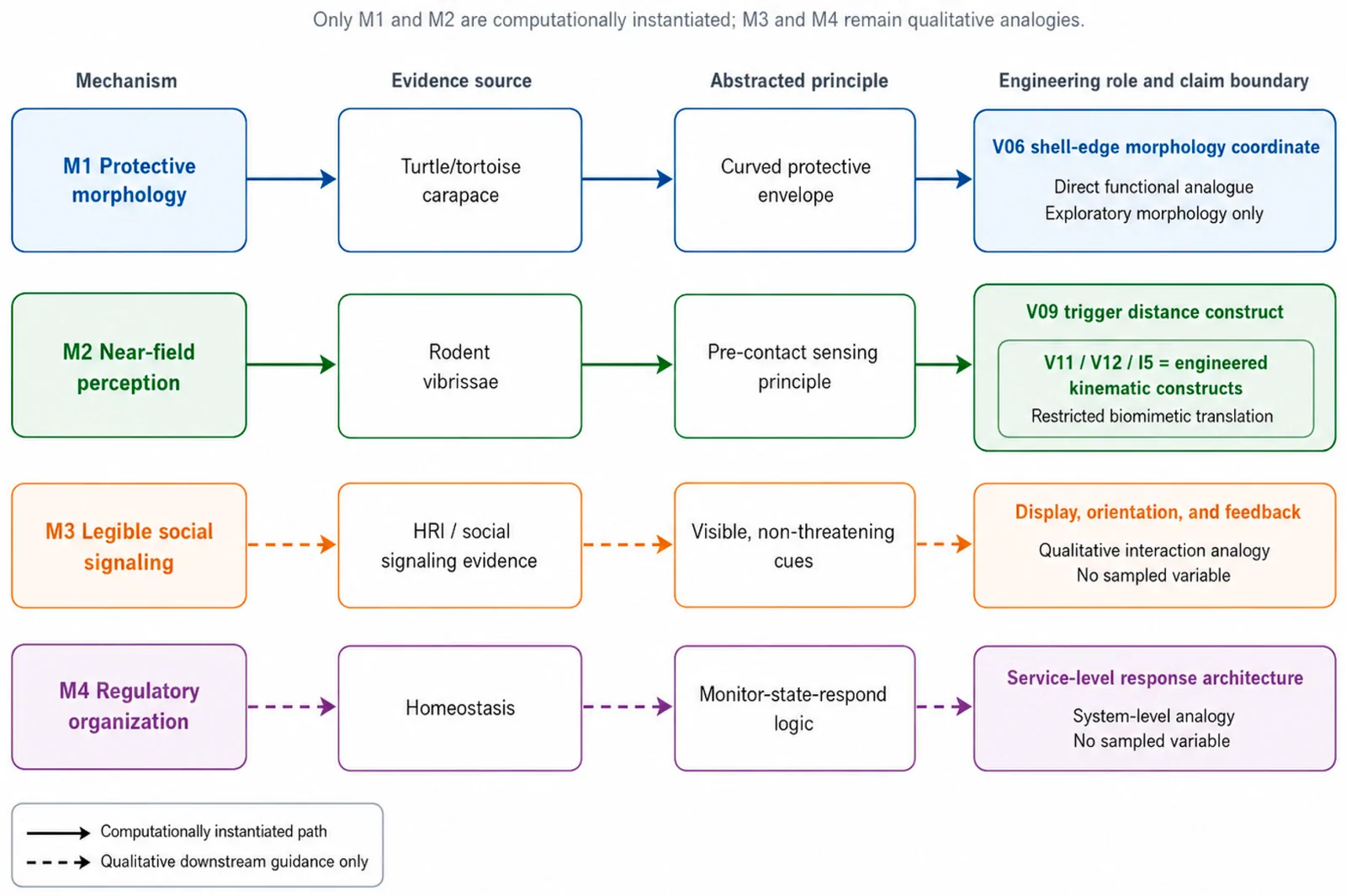
Evidence-tiered relationships among the four biomimetic mechanisms. M1 contributes V06 as an exploratory morphology coordinate. M2 contributes V09 as a pre-contact trigger distance construct, while V11, V12, and I5 remain engineered kinematic constructs rather than biologically validated metrics. M3 and M4 guide qualitative downstream interaction and service-organization decisions and contribute no sampled variables. (Author’s own work).

Figure 2 separates four levels that should not be conflated: evidence source, abstracted function, computational role, and downstream engineering role. M1 supports only the translation of protective envelope morphology into V06 as an exploratory shell-edge coordinate. M2 supports a pre-contact sensing analogy through V09, whereas V11 and V12 are study-defined engineering inputs and I5 is a derived kinematic proxy rather than a biologically validated metric. M3 guides qualitative interface, feedback, and signaling decisions, and M4 guides the conceptual organization of monitoring, state evaluation, and response adjustment. M3 and M4 contribute no sampled variables or numerical scores. Ordinary stability, ergonomic, emergency stop, material, and manufacturing decisions remain outside the biomimetic computation. The evidence-tiered source–principle–translation relationships and their operationalization status are summarized in Table 1.

**Table 1:** Evidence-tiered source–principle–translation matrix and operationalization status.

No.	Mechanism	Biological Source	Functional Principle	Product-Level Translation	Operationalization Status	Evidence
1	M1 Direct functional analog	Turtle/tortoise carapace	Curved, layered protective envelope	Nominal external-shell-edge treatment	V06 exploratory morphology coordinate	Achrai and Wagner 2017 [16]
2	M1 Downstream implementation	No additional biological source asserted	Compliant contact as an engineering follow-on to protective intent	Material, thickness, hardness, attachment, and cleanability	Downstream; not computationally screened	Requires material and contact testing
3	M2 Direct functional analog	Rodent vibrissae	Pre-contact sensing	Longitudinal pre-braking trigger zone	V09 exploratory trigger distance coordinate	Wei et al. 2022 [17]
4	M2 Engineered response coupling	Vibrissa analogy retained at sensing-intent level	Detection-to-response coupling	Pre-braking speed, commanded deceleration, and warning margin	V11/V12 and derived I5	Reference [17] supports sensing analogy; kinematics are study-defined
5	M3 Qualitative interaction analogy	No direct biological source claimed	Legible, non-threatening feedback	Display and feedback placement	Qualitative downstream; no sampled metric	HRI evidence [13,18,19]
6	M4 System-level analogy	Homeostatic regulation	Monitoring, state evaluation, and adaptive response	Service-level sensing–response architecture	Qualitative; no sampled metric	Cannon 1932 [22]
7	Engineering stability consideration	Support-base and component layout	Passive stability through support polygon and mass distribution	Base width and post-layout COG verification	V04/V05 downstream; not biomimetic	Engineering verification required
8	Safety interface consideration	Human factors and safety standards	Accessible emergency stop	Emergency stop placement	V10 downstream; not biomimetic	ISO 13482 qualitative requirement [35]

#### 3.2.1. M1—Protective Morphology and Downstream Compliant-Contact Intent

The turtle and tortoise carapace provides a documented analog for a curved, layered protective envelope [16]. The present computation translates only the shell-edge aspect into V06, a nominal external-shell-edge radius coordinate. Compliant materials, thickness, hardness, attachment, and cleanability remain downstream engineering specifications and are not separately claimed as validated biomimetic variables.

#### 3.2.2. M2—Pre-Contact Sensing Analogy and Engineered Graduated Response

Rodent vibrissae provide a documented pre-contact sensing analog [17]. V09 is an engineered longitudinal trigger distance translation of that intent. V11, V12, the braking distance equation, and I5 are study-defined kinematic constructs; the biological source does not validate their numerical bounds or physical stopping performance. Fish lateral-line and insect antenna claims were removed because the cited evidence did not directly support them.

#### 3.2.3. M3—Qualitative Affiliative Signaling and Legible Feedback

M3 is retained as a qualitative interaction analogy, not as a claimed biological mechanism with a numerical metric. Human–robot interaction studies support the relevance of friendly, legible feedback for trust and acceptance [13,18,19]. Display center height, orientation, and feedback light placement are therefore downstream ergonomic decisions requiring user evaluation [20,21].

#### 3.2.4. M4—Homeostatic Regulation as a System-Level Analogy

Homeostasis provides an architectural analogy for continuous monitoring and adaptive response [22]. M4 contributes no sampled variable or composite score. It guides only the conceptual organization of sensing, state evaluation, and response adjustment; system effectiveness would require implementation and testing.

### 3.3. Candidate-Variable Audit and Final Computational Scope

**Figure 3:**
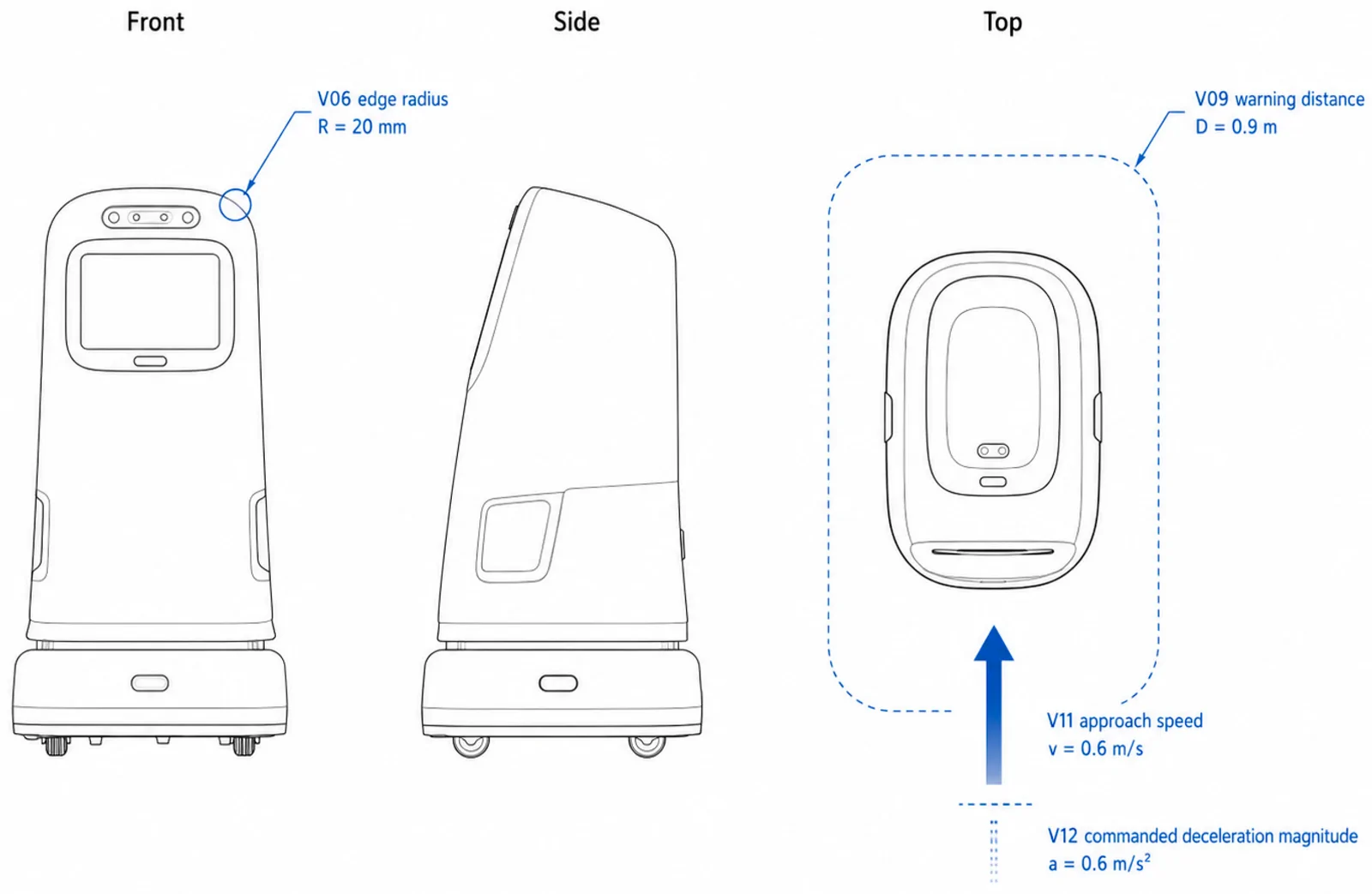
Four-dimensional computational design space and B9 braking distance proxy constraint. V06 is an exploratory morphology coordinate; V09, V11, and V12 form the coupled kinematic subspace. The model excludes latency and physical validation. (Author’s own work).

The initial formulation contained 15 continuous variables. The revision audit removed variables that were undefined, unsupported, duplicated another construct, disconnected from the evaluation, or more appropriately set during downstream CAD and engineering development. The final computation retains V06, V09, V11, and V12; the remaining candidate parameters are reported transparently as non-screened implementation, ergonomic, safety interface, scale, or post-layout engineering decisions (Table 2). Figure 3 shows the final four-dimensional scope.

### 3.4. Exploratory Ranges and Evidence Classification

The final ranges were frozen before execution: V06 = 10–80 mm, V09 = 0.5–1.5 m, V11 = 0.2–1.0 m/s, and V12 = 0.2–0.8 m/s^2^. They are study-defined exploratory scenario bounds rather than validated operating limits. V11 is partly anchored by published maximum translational speeds of service-robot products, but maximum product speed is not equivalent to a human-approach speed. The V09 and V12 bounds are author-defined assumptions. Different bounds would change the geometry of the feasible domain. Different bounds would change the geometry of the feasible domain (Table 3).

**Table 3:** Final four-dimensional design space specification, derived proxy, and diagnostic protocol.

Parameter	Definition	Value or Range	Unit	Status	Interpretation
V06	Nominal external-shell-edge radius	10–80	mm	Sampled	Exploratory morphology coordinate
V09	Longitudinal pre-braking trigger distance	0.5–1.5	m	Sampled	Distance at which controlled deceleration begins
V11	Pre-braking translational speed	0.2–1.0	m/s	Sampled	Commanded speed immediately before braking
V12	Constant commanded deceleration magnitude	0.2–0.8	m/s^2^	Sampled	Simplified braking-model input
d_brake_	V11^2^/(2V12)	Derived	m	Proxy	Uniform-deceleration braking distance
I5	1 − d_brake_/V09	0–1	—	Derived proxy	Normalized kinematic warning margin
B9	V09 − d_brake_ ≥ 0	Hard constraint	m	Mandatory	Rejects infeasible candidates without repair
P0	10 seeds × 10,000 candidates	100,000	candidates	Pre-check	Feasibility and I5 information gate
CFRS	Common feasible reference set	16,384	points	Reference only	Coverage and stability diagnostics
N sequence	Adaptive formal sample sizes	32–1024	points	Pre-specified	Ten levels; fixed seeds
Distances	d_A_ primary; d_B_ sensitivity	Fixed-range normalized	—	Pre-specified	I5 excluded from distance space
m candidates	Maximin representative count	2–5	points	Pre-specified	Plateau rule; no natural-type claim

Note: The numerical ranges in Table 3 define the computational study domain only. They do not establish elderly-specific comfort thresholds, certified safety distances, or standard-compliant braking performance.

ISO 13482 is used only as qualitative background for shape-hazard mitigation and the need for safety verification [35]. It is not cited as the source of the V06, V09, or V12 numerical bounds. ISO 7176-6 and related test documents may inform future measurement protocols, but their wheelchair test conditions do not directly validate the present service-robot deceleration range [36,37,38].

### 3.5. Constraint-Based Four-Dimensional Design Space Exploration

Latin hypercube sampling was used to generate candidates across the four locked ranges [31,32]. Each candidate was expressed in fixed-range normalized coordinates for distance and coverage calculations. V06 remained independent of the kinematic feasibility filter; V09, V11, and V12 were coupled by the B9 constraint.

The braking distance proxy was defined as d_brake_ = V11^2^/(2V12). A candidate was feasible only when g(x) = V09 − d_brake_ ≥ 0. Infeasible candidates were discarded without clipping, repair, or silent parameter adjustment. Because sensing, processing, control, and actuator latency were unavailable, d_brake_ is not an actual stopping distance or certified protective distance.

### 3.6. Feasibility Pre-Check and Normalized Kinematic Warning Margin

Before the adaptive sample size study, P0 generated 10,000 four-dimensional LHS candidates for each of 10 fixed seeds. The pre-specified gates required a pooled feasible acceptance rate of at least 0.10, a seed-level acceptance-rate standard deviation no greater than 0.03, and an I5 P95–P05 spread of at least 0.05.

#### Derived Physics-Informed Proxy

For each feasible candidate, the normalized kinematic warning margin was calculated as I5 = 1 − d_brake_/V09.

I5 is bounded within [0, 1] by the B9 constraint. Values near 1 indicate a low braking distance demand relative to the trigger distance, whereas values near 0 indicate that the braking distance proxy approaches the full trigger distance.

I5 is not a collision safety score. It excludes system latency, surface friction, moving pedestrians, actuator saturation, controller dynamics, and measured physical stopping behavior.

### 3.7. Adaptive Sample Size and Coverage Diagnostics

Formal constrained samples were evaluated at N ∈ {32, 64, 96, 128, 192, 256, 384, 512, 768, 1024}. Ten fixed seeds were used for initial screening, and 30 fixed seeds were used for the final verification boundary. For each N and seed, at least 10N feasible candidates were generated before a deterministic space-filling subset was selected.

C1 assessed paired changes in I5 mean, median, standard deviation, P05, and P95. C2 assessed the empirical I5 distribution using the two-sample Kolmogorov–Smirnov statistic. Both diagnostics used pre-specified study-specific thresholds and paired seed comparisons.

C3 assessed coverage relative to a fixed constrained feasible reference set (CFRS) of 16,384 feasible points. Mean and P95 nearest-sample distances were calculated in fixed-range normalized space, and the P05, P50, and P95 of each of the four variables were compared with the CFRS. All variable quantiles were normalized before application of the 0.05 absolute-difference threshold.

A sufficient N required two consecutive N levels to satisfy all mandatory diagnostics under the staged 10-seed/30-seed rule. These thresholds were pre-specified for this study and are not universal LHS sample size rules.

Complete pre-specified definitions, numerical thresholds, seed-aggregation rules, pass/fail criteria, and failure actions for P0 and C1–C7 are provided in Supplementary Table S1.

### 3.8. Diversity-Based Configuration Selection and Robustness Checks

A deterministic greedy maximin procedure selected diverse points from each formal sample. The primary distance d_A_ was dimension-normalized Euclidean distance across the four fixed-range normalized variables. Candidate representative counts m = 2–5 were compared, and the smallest m at the pre-specified coverage-improvement plateau was retained [32].

Before cluster-derived interpretation, C4 evaluated cluster tendency using the Hopkins statistic, the gap statistic, and silhouette width [33,34]. If the gate failed, no cluster types were reported and maximin diversity selection remained the only configuration-selection procedure.

C6 evaluated cross-seed coordinate stability by Hungarian matching of maximin-selected sets. C7 compared the primary equal-variable distance with a morphology-versus-kinematics block-balanced distance. C7 was a reporting-only sensitivity analysis and did not alter sample size sufficiency.

The primary distance was d_A_ = [(Δz06^2^ + Δz09^2^ + Δz11^2^ + Δz12^2^)/4]½. The alternative distance assigned one-half of the total contribution to V06 and one-half jointly to V09, V11, and V12. I5 was excluded from both distance spaces because it is derived from three of the sampled variables.

The procedure did not generate an aggregate benefit score, a TOPSIS rank, or a globally optimal alternative. When exact coordinates were unstable, fixed-seed outputs were retained only as illustrative boundary examples.

#### Validity, Reliability, and Reporting Scope of the Diagnostic Indicators

The diagnostic indicators were defined to assess internal properties of the computational exploration rather than physical robot safety, usability, or product performance. Construct validity was addressed by assigning each diagnostic to one delimited methodological question. P0-A evaluates feasible-domain yield, whereas P0-B evaluates the information spread of the derived I5 proxy. C1 and C2 evaluate the descriptive and distributional stability of I5. C3 evaluates coverage of the constrained four-dimensional domain. C4 tests whether a detectable natural cluster structure exists. C6 evaluates the cross-seed coordinate stability of maximin-selected configurations, and C7 evaluates sensitivity to the distance definition. The indicators therefore assess the behavior, stability, and reporting limits of the computational procedure; they do not validate an elderly-care robot as a physical product.

Computational reliability was addressed through locked variable definitions and ranges, pre-specified thresholds, fixed seed lists, repeated paired-seed comparisons, consistent aggregation rules, and explicit pass/fail and hard-stop criteria. The successful C1 and C2 outcomes support stability of the I5 distribution within the specified computational setting. Conversely, the failure of C3, the non-activation of C4, the failure of C6, and the sensitivity detected by C7 demonstrate that the protocol can disclose instability rather than force a positive convergence result.

External criterion validity and independent computational reproducibility were not established because the study did not include a physical prototype, measured sensing and actuator latency, empirical stopping tests, human-subject evaluation, raw sampled configurations, or a source-code package. Accordingly, all indicators are reported as study-specific diagnostics or proxies. Supplementary Table S1 reports their complete definitions, pre-specified thresholds, seed-aggregation rules, failure actions, hard stops, and observed summary outcomes. A summary of the intended constructs, construct-validity basis, reliability assessment, and claim limitations of the diagnostic indicators is provided in Table 4.

**Table 4:** Construct validity, computational reliability, and claim scope of the diagnostic indicators.

Diagnostic Group	Intended Construct	Construct-Validity Basis	Reliability Assessment	Claim Limitation
P0-A/P0-B	Feasible-domain yield and I5 information spread	Directly defined from B9-filtered candidates and the observed I5 distribution	Ten fixed seeds, pooled statistics, and pre-specified gates	Does not establish physical safety or operating suitability
C1/C2	I5 descriptive and distributional stability	Paired descriptive-statistic and empirical-CDF comparisons	Thirty paired seeds at the final verification boundary	Applies only to stability of the derived proxy distribution
C3	Four-dimensional feasible-space coverage	CFRS-based nearest-sample distances and normalized variable-quantile comparisons	Thirty paired seeds and fixed-range normalization	Failed; full design space coverage stability was not established
C4	Detectable natural cluster structure	Hopkins, Gap-statistic, and Silhouette gate	Repeated assessment across the fixed seeds	Gate failed; no cluster-derived design types were reported
C6/C7	Coordinate stability and distance definition sensitivity	Hungarian matching, Hausdorff distance, and comparison of alternative distance definitions	Thirty fixed seeds and pre-specified thresholds	C6 failed and C7 was sensitive; no stable representative solutions were claimed

### 3.9. Design-Strategy Translation

The computational outputs were translated through a complete strategy matrix rather than an automated optimization rule. V06 informed shell-edge morphology; V09, V11, V12, and I5 informed the conceptual relation between trigger distance, approach speed, and commanded deceleration. M3, M4, ergonomics, material behavior, interface placement, CMF, packaging, and manufacturing remained downstream decisions.

The earlier 16-alternative ranking table was removed because it depended on invalidated criteria and scoring. The revised constrained space is continuous and did not yield stable natural clusters; fabricating a finite matrix of “all concepts” would therefore require arbitrary discretization. Table 5 reports the complete set of downstream strategies actually used, while the two visual directions are explicitly illustrative rather than exhaustive or computed winners.

**Table 5:** Post-screening product design translation matrix.

ID	Strategy	Biomimetic Mechanism	Computational Input	Product Design Translation	Claim Boundary
S1	Shell-edge morphology	M1	V06	Rounded or tighter external-shell transitions	Morphology only; not a safety score
S2	Compliant-contact implementation	M1	None	Material, thickness, hardness, attachment, and cleanability	Qualitative downstream; not screened
S3	Pre-braking trigger zone	M2	V09	Longitudinal zone for initiating controlled deceleration	Not a certified protective distance
S4	Speed deceleration response	M2	V11, V12, I5	Conceptual relation between approach speed and braking demand	No latency or physical stopping validation
S5	Affiliative interface and signaling	M3	None	Legible interface, visible feedback, non-threatening cues	Requires ergonomic and user evaluation
S6	Adaptive service-response architecture	M4	None	Monitoring, state evaluation, and response adjustment	System-level analogy; not a computed score

### 3.10. AI-Assisted Concept Visualization: Procedure and Provenance

AI-assisted visualizations were used only to communicate two qualitative product design directions. Project records indicate that the prompts specified the elderly-care context, broad morphology, interface role, CMF intent, domestic setting, and exclusion instructions. These records predate the revised four-dimensional computation and include legacy design labels; they are therefore provenance information rather than executable specifications for ILL_B01_ or ILL_B02_. Project records identify OpenAI GPT-4o, but complete prompt histories, exact model-build metadata, generation settings, output counts, selection logs, and post-processing histories were not retained and are not included in the Supplementary Materials. The images cannot be exactly reproduced from the available record and are not scientific evidence.

### 3.11. Rhino-Based Conceptual CAD: Modeling Scope and Limitations

The Rhino files are legacy conceptual volume studies used to organize shell, base, interface, and service–component relationships. Their scripts retain superseded alternative labels and parameters, so the models are not presented as direct realizations of the revised D2 coordinates. They do not include mass properties, internal packaging, actuator mounting, structural analysis, tolerances, or manufacturing details.

The Rhino and Grasshopper source files are not included in the Supplementary Materials. The retained figures should therefore be interpreted as qualitative conceptual-volume documentation rather than independently reproducible CAD deliverables.

### 3.12. Data Availability and Ethical Scope

The sole Supplementary File provided with this article is Supplementary Table S1. It reports the pre-specified diagnostic definitions, thresholds, seed-aggregation rules, pass/fail criteria, failure actions, hard stops, and observed summary outcomes for P0 and C1–C7. Source code, raw sampled configurations, intermediate diagnostic outputs, implementation-correction records, complete AI prompts, and Rhino or Grasshopper source files are not included in the Supplementary Materials. The numerical findings should therefore be interpreted as summary-level computational outputs documented in the manuscript and Supplementary Table S1. No experimental, survey, clinical, or user-study data were generated.

The study did not involve human participants, personal data, human tissue, clinical intervention, animal experimentation, or physical testing with older adults. Institutional review board approval was therefore not sought; this determination remains subject to the authors’ institutions and the journal’s requirements.

## 4. Results

### 4.1. Biomimetic Mapping and Variable-Scope Reduction

The evidence-tiered audit retained M1 and M2 as direct functional analogs at delimited levels, M3 as a qualitative interaction analogy, and M4 as a system-level analogy (Figure 2; Table 1). Only M1 and M2 were computationally instantiated: M1 through V06 as an exploratory shell-morphology coordinate and M2 through V09, V11, V12, and the derived I5 proxy.

Of the 15 initial candidate variables, four entered the final computational space. The remaining variables were reassigned to post-screening scale, footprint, mass-property, compliant-contact, safety interface, ergonomic, signaling, or service-architecture decisions (Table 2). This separation removed undefined coverage ratios, unverified anthropometric scoring functions, duplicate stability constructs, and variables that did not affect the original ranking.

### 4.2. P0 Feasibility and Feasible-Domain Characterization

Across 100,000 P0 candidates, 86,212 satisfied B9, giving a pooled acceptance rate of 0.8621 (Wilson 95% CI: 0.8600–0.8642). The seed-level acceptance-rate standard deviation was 0.0016. I5 had a mean of 0.6319, median of 0.6868, IQR of 0.4010, P05 of 0.1300, and P95 of 0.9477; the P95–P05 spread was 0.8177. P0 therefore passed all three pre-specified gates.

The feasible samples retained the full marginal range of each variable, although the joint V09–V11–V12 domain was cut by B9. As expected from the formula, V11 was strongly positively associated with d_brake_ and negatively associated with I5, while V06 remained approximately independent of the kinematic variables. These associations describe the imposed model structure and are not causal evidence from a physical robot.

### 4.3. I5 Distributional Stability

**Figure 4:**
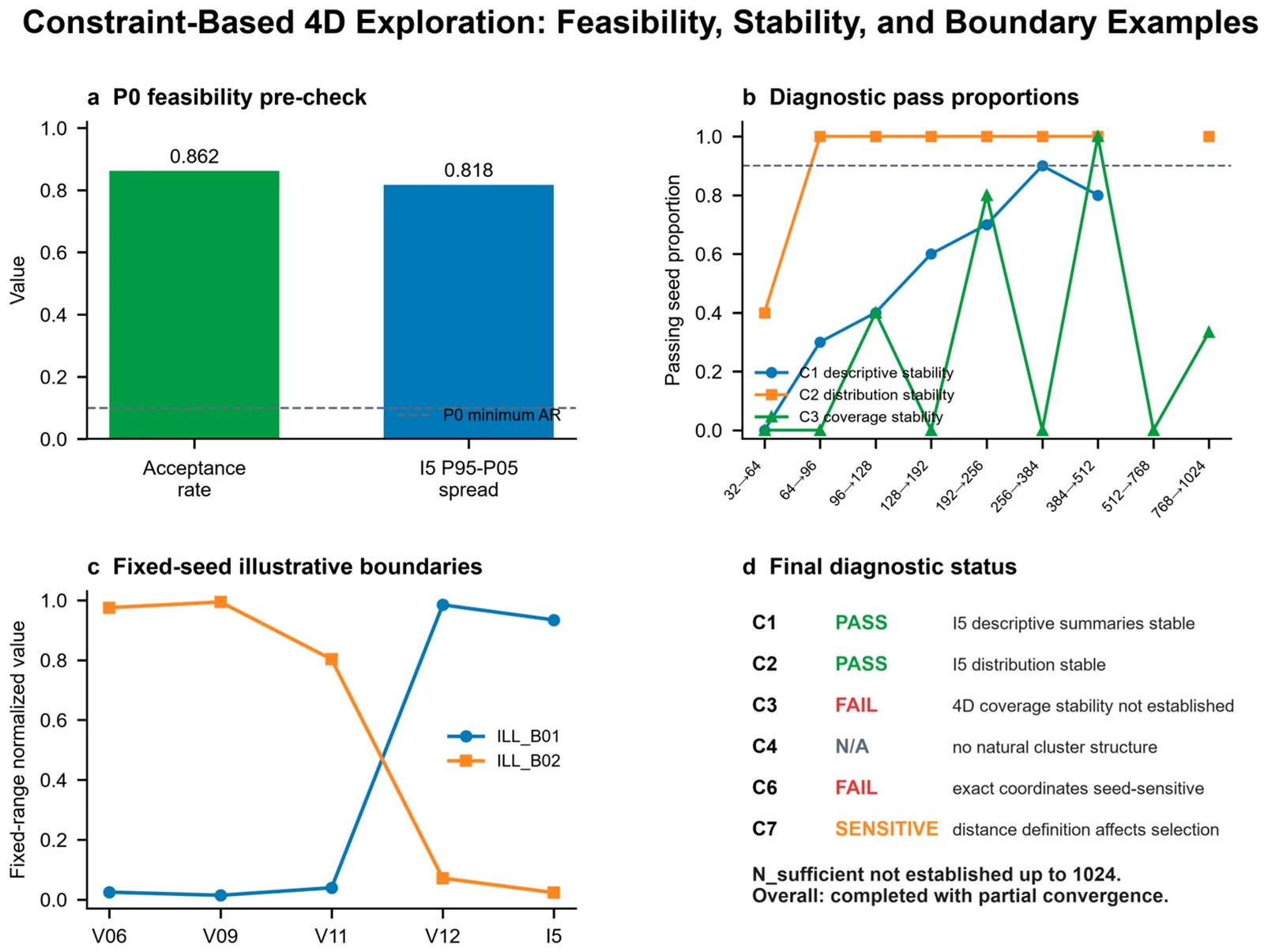
Feasibility, stability, coverage, and illustrative-boundary diagnostics. Panel (**a**) summarizes P0; panel (**b**) shows C1–C3 seed pass proportions; panel (**c**) compares the two fixed-seed boundary examples; panel (**d**) reports the final diagnostic status. Values for the examples were computed from full-precision coordinates before display rounding. (Author’s own work).

C1 improved with sample size and passed the 30-seed verification at N = 768→1024, with all 30 paired seeds satisfying the five descriptive-statistic limits. C2 passed from N = 64→96 onward in the 10-seed screening and passed at N = 768→1024 for all 30 paired seeds; the median KS statistic was 0.0249. The corresponding feasibility, stability, coverage, illustrative-boundary, and final diagnostic results are summarized in Figure 4.

These results show that the distribution of the derived I5 proxy was stable under the repeated-seed protocol at the largest evaluated sample pair. They do not establish stability of the full four-dimensional coverage or of individual selected coordinates.

### 4.4. Coverage Stability and Sample Size Sufficiency

C3 was recalculated after correcting an implementation error in which raw-unit quantile differences had been compared directly with a normalized threshold. With fixed-range normalization applied, only 10 of 30 seeds passed the combined C3 rule at N = 768→1024. The largest reported normalized quantile deviation was 0.0494 at P95 of V11; additional failures arose from the full set of distance and quantile conditions.

No consecutive N pair satisfied all mandatory distributional, coverage, and representative-set conditions. Consequently, N_sufficient_ was not established up to the maximum evaluated N of 1024. N = 1024 is reported only as the maximum sample size tested, not as a universally sufficient or fully converged design space sample.

### 4.5. Cluster Tendency

The cluster tendency gate did not pass: the median Hopkins statistic was 0.53, the proportion of seeds for which the gap statistic selected k ≥ 2 was 0.40, and the median k = 2 silhouette width was 0.18. No natural cluster structure was therefore reported, and the conditional cluster-based pathway was not used.

### 4.6. Illustrative Boundary Configurations

The pre-specified coverage-improvement plateau occurred at m = 2. Deterministic maximin selection from the fixed maximum-size run at N = 1024 and seed 2026072501 produced two widely separated illustrative boundary configurations (d_A_ = 0.906).

ILL_B01_ combined V06 = 11.73 mm, V09 = 0.514 m, V11 = 0.232 m/s, and V12 = 0.791 m/s^2^, yielding d_brake_ = 0.034 m and I5 = 0.934. It illustrates a low-speed, high-deceleration region with a large normalized warning margin under the simplified model.

ILL_B02_ combined V06 = 78.26 mm, V09 = 1.494 m, V11 = 0.842 m/s, and V12 = 0.243 m/s^2^, yielding d_brake_ = 1.460 m and I5 = 0.023. It illustrates a configuration close to the B9 boundary. This low margin must not be interpreted as a preferred performance direction.

### 4.7. Representative-Set and Distance Definition Stability

C6 failed despite successful implementation validation. Across the 30 fixed seeds, the median mean-matched distance was 0.4515, the P90 mean-matched distance was 0.6369, and the median Hausdorff distance was 0.4718, all exceeding the pre-specified thresholds. Exact maximin-selected coordinates were therefore seed-sensitive, and H5 (UNCERTAIN_REPRESENTATIVE_SET) was triggered.

A supplementary profile analysis also showed substantial coordinate and qualitative-profile variability across seeds. Although the high-I5 member had lower V11 than the low-I5 member in 96.7% of seeds, the remaining variables did not form a stable two-type pattern.

C7 also indicated sensitivity to distance definition: the overlap between representative sets selected using the equal-variable distance and the block-balanced distance was 0.53, below the pre-specified 0.75 threshold.

The final D2 status was therefore “completed with partial convergence”: I5 distributional stability was established, whereas feasible-space coverage, natural clusters, exact representative coordinates, and distance definition robustness were not.

### 4.8. Product Design Translation Outcomes

**Figure 5:**
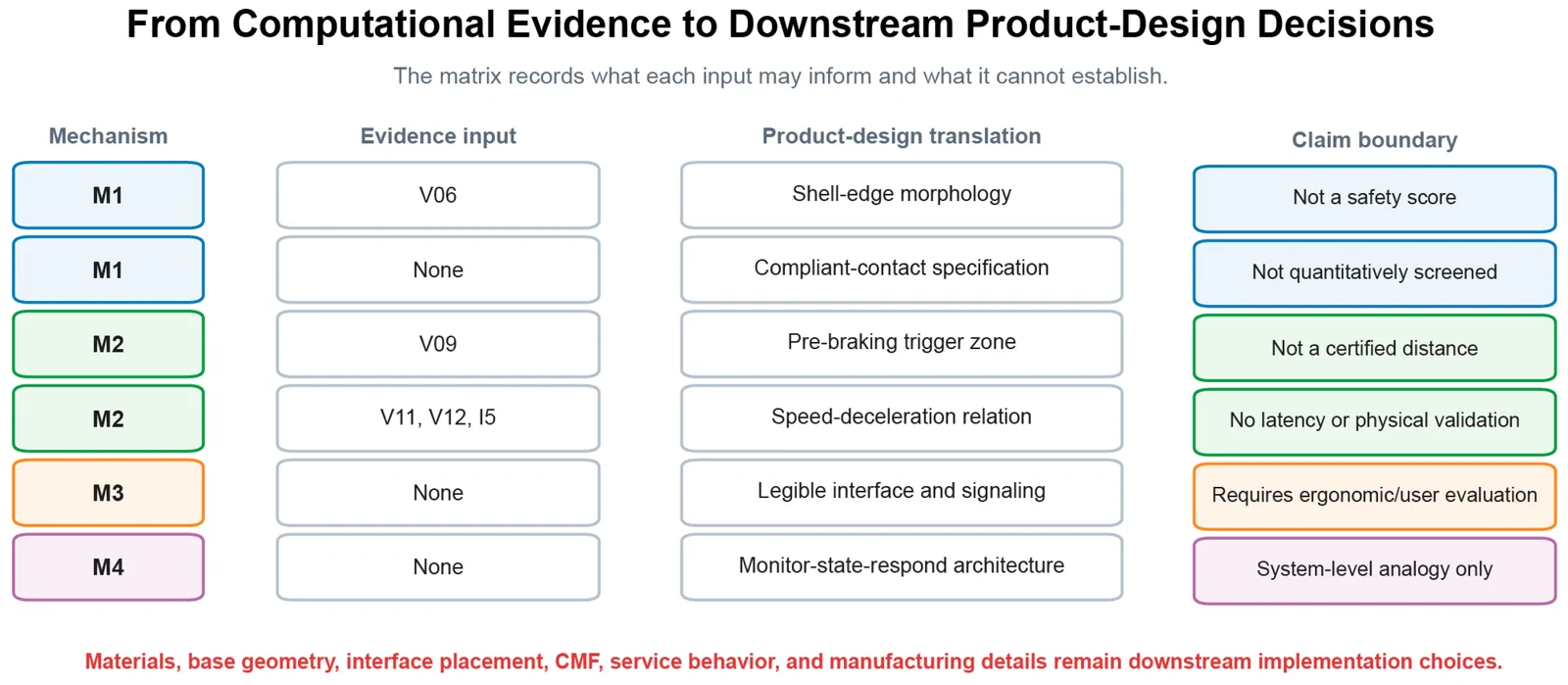
Evidence-to-design translation matrix. Computational coordinates inform only the delimited roles shown; ergonomics, materials, interface placement, service behavior, and manufacturing remain downstream. (Author’s own work).

Because neither an optimal ranking nor stable representative coordinates were established, the design translation was organized as a set of downstream decisions rather than as two computed “winning” concepts. Table 5 and Figure 5 separate computational inputs from qualitative implementation choices.

Concept Direction A emphasizes a rounded, low-threat product language, a calm interface, and compliant-contact intent. These elements arise primarily from the qualitative M1 and M3 design principles and are not a direct geometric realization of ILL_B01_.

Concept Direction B emphasizes a service-oriented body, a more explicit interface, and visible operational cues. These elements illustrate an alternative product language but are not validated by the low-margin ILL_B02_ coordinates.

The visual and CAD outputs therefore demonstrate how the framework can support design communication after computational exploration. They do not resolve the uncertainty in the selected coordinates and are not used to claim safety, usability, or superiority.

### 4.9. AI-Assisted Product Concept Visualization

**Figure 6:**
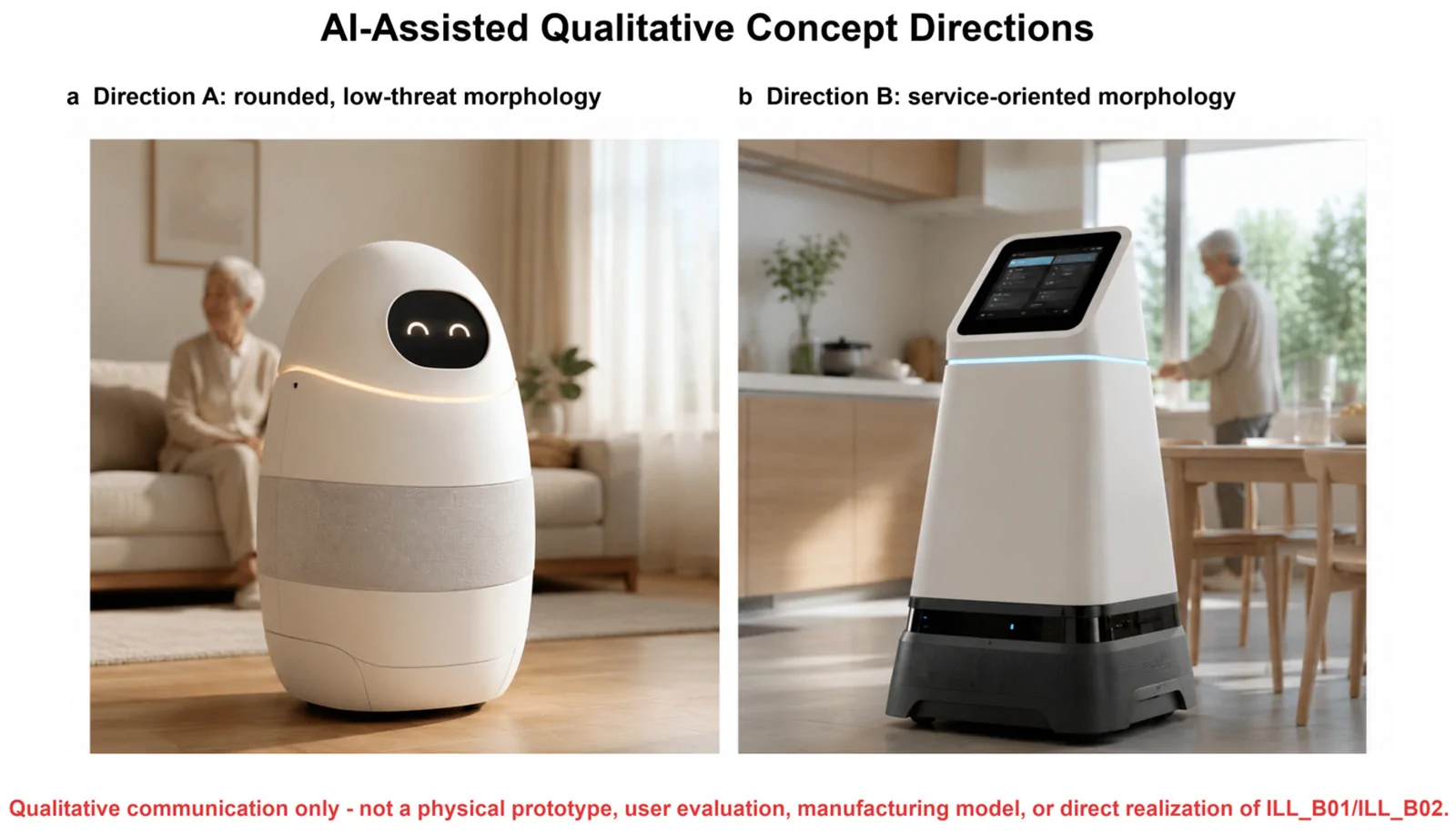
AI-assisted qualitative concept directions. The renderings are legacy design communication artifacts and are not direct realizations of the seed-sensitive boundary coordinates. (Author’s own work).

Figure 6 presents two AI-assisted qualitative concept directions. They communicate broad morphology, interface emphasis, CMF, and domestic context. Because the available generation record is incomplete and predates D2, the images are not treated as reproducible outputs, direct realizations of ILL_B01_/ILL_B02_, physical prototypes, or user-evaluation evidence.

### 4.10. Rhino-Based Conceptual CAD

**Figure 7:**
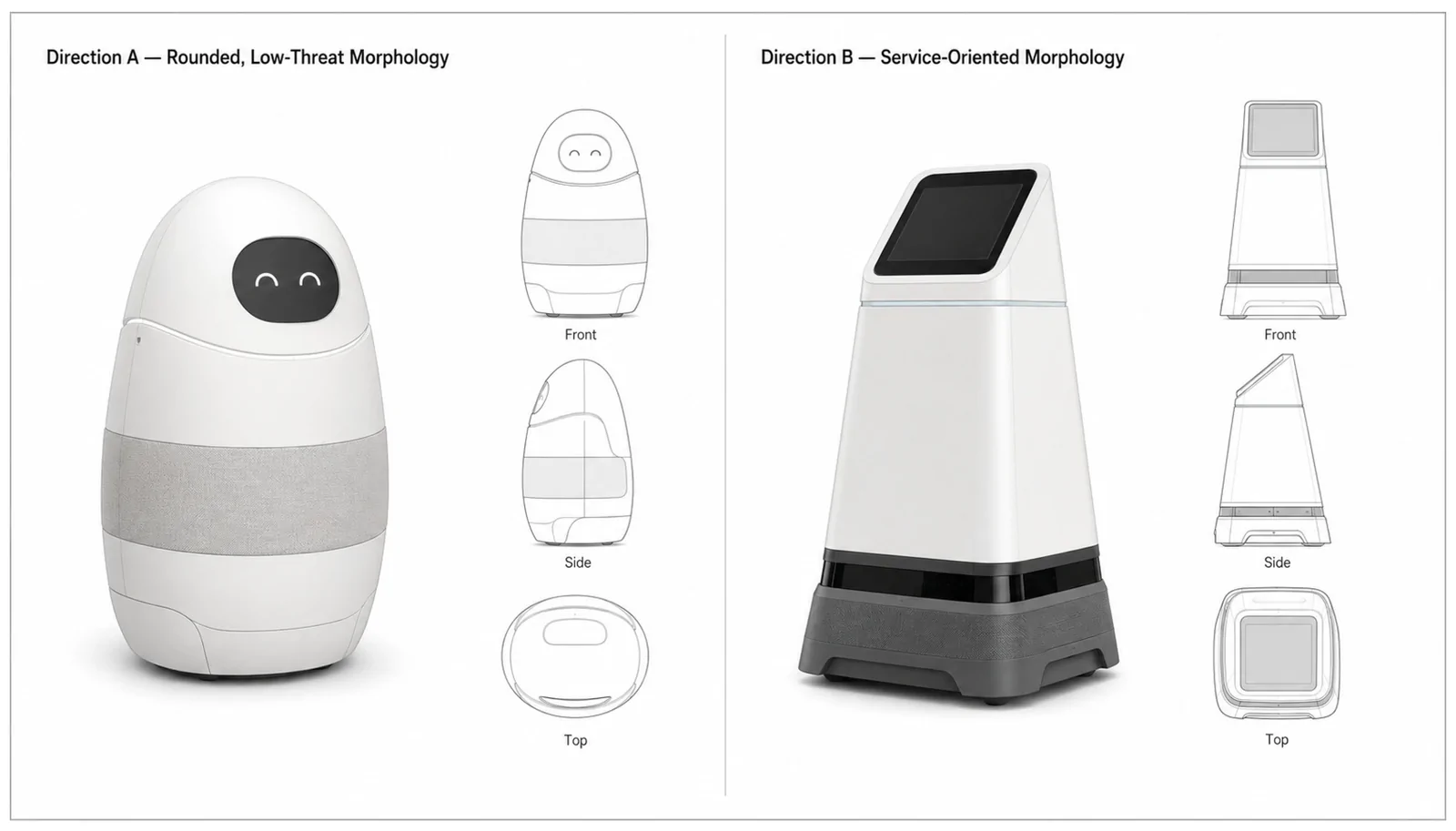
Two downstream conceptual CAD directions developed from the revised four-variable exploratory framework. Direction A emphasizes a rounded, low-threat morphological language, whereas Direction B emphasizes a more service-oriented product architecture. The views communicate downstream conceptual design directions rather than ranked, statistically stable, validated, or optimal robot solutions. The displayed overall dimensions were determined during downstream CAD development and were not computationally screened. (Author’s own work).

Figure 7 shows two downstream conceptual CAD directions developed from the revised four-variable exploratory framework. Direction A emphasizes a rounded, low-threat morphological language, whereas Direction B emphasizes a more service-oriented product architecture; the views communicate conceptual directions rather than ranked, statistically stable, validated, or optimal robot solutions.

### 4.11. Integration and Claim Boundary

Together, Figure 5, Figure 6 and Figure 7 show how the constrained computational exploration is translated into downstream product design communication. Figure 5 identifies the delimited computational inputs and the decisions that remain downstream, whereas Figure 6 and Figure 7 present qualitative visualization and conceptual CAD directions. These outputs illustrate morphology, interface, CMF, and layout hypotheses only; they do not demonstrate physical safety, manufacturability, elderly-user acceptance, empirical superiority, or stable representative-solution convergence. Table 6 summarizes the final diagnostic status and the corresponding reporting limitations.

## 5. Discussion

### 5.1. Contribution to Product-Level Biomimetic Design

The evidence-tiered framework extends biomimetic reasoning to product-level morphology, approach behavior, signaling, and service logic while making the evidence boundary explicit. Biological sources are not treated as decorative motifs or as direct validators of engineering performance. Instead, each source is linked to a delimited translation role and an explicit validation need.

The reduction from 15 candidate variables to four computational variables is itself an important result. It shows that a traceable biomimetic workflow may become more credible by removing weakly defined variables rather than by maximizing parameter count. M1 is represented by a single exploratory shell-edge coordinate, M2 by a coupled kinematic subspace, M3 by qualitative interaction guidance, and M4 by a system-level analogy.

This evidence-tiered structure avoids two recurrent problems in product-level biomimetics: attributing ordinary engineering requirements to biological sources and presenting qualitative intentions as validated numerical metrics. It also makes the computational asymmetry transparent: M1 and M2 are operationalized only at restricted levels, whereas M3 and M4 remain qualitative.

### 5.2. From Aggregate Ranking to Constrained Exploration

The original formulation used objective weighting and TOPSIS. The audit showed that several criteria were unvalidated, duplicated another construct, or had no defensible countervailing objective. Entropy and CRITIC can characterize dispersion and statistical conflict [25,26], and TOPSIS can aggregate a justified decision matrix [27,28,29], but these methods cannot validate the underlying criteria. Their removal prevents a data-dependent weighting scheme from converting exploratory author preferences into an apparently objective optimum.

Constraint-based exploration is better aligned with the available evidence. B9 excludes combinations that violate a transparent closed-form relation, while the remaining outputs describe feasibility, distributional stability, coverage, and coordinate sensitivity. The approach is therefore a front-end mapping procedure rather than a performance optimizer.

### 5.3. Interpretation of Partial Convergence

C1 and C2 show that the distribution of I5 became stable at the largest evaluated pair. C3 and C6 show that this distributional stability did not extend to full coverage or exact representative coordinates. This distinction is substantive: a stable one-dimensional derived proxy does not imply that a four-dimensional design space has been fully characterized.

The absence of natural clusters and the sensitivity to distance definition further indicate that the feasible domain is better interpreted as a continuous constrained space than as a set of discrete design types. Maximin selection consistently improves geometric coverage, but with m = 2 it favors distant boundary points whose exact locations depend on the finite candidate pool. Reporting this instability is more informative than retrospectively increasing m or relaxing thresholds to obtain a nominal pass.

### 5.4. Implications for Product Design Translation

V06 should be interpreted as a morphology coordinate, not as an edge-safety score. A larger nominal radius may create a rounder shell transition, but its visual and contact effect depends on final robot scale, adjacent geometry, material, and manufacturing process. These considerations must be checked during CAD development.

V09, V11, and V12 provide a transparent relation between trigger distance, pre-braking speed, and commanded deceleration. I5 helps compare braking demand within the simplified domain, but it does not include latency or measured robot dynamics. ILL_B02_ demonstrates why feasibility alone is insufficient: a point may satisfy B9 while lying very close to the modeled boundary.

M3 and M4 remain useful even without numerical scores. Legible signaling, non-threatening interaction language, and adaptive service logic can organize downstream design decisions, but they require ergonomic analysis, user testing, and system implementation before performance claims are possible. The AI and CAD outputs should therefore be read as visual hypotheses for future evaluation.

### 5.5. Limitations

First, all four numerical ranges are study-defined exploratory bounds. They were not derived from a formal requirements analysis or stakeholder survey, and alternative bounds would change the feasible-domain geometry and the location of illustrative examples.

Second, the braking distance proxy assumes immediate detection and commanded deceleration. Real systems include sensing, processing, communication, control, and actuator latency, so the effective stopping distance would be d_total_ = v·t_latency_ + v^2^/(2a). No latency term was introduced because an authenticated end-to-end value was unavailable.

Third, B9 and I5 were not validated against a physical robot, hardware-in-the-loop system, measured friction condition, moving pedestrian, or actual stopping distance test. ISO 13482 and related test guidance remain future verification references rather than evidence for the present numerical bounds [35,36,37,38].

Fourth, sample size sufficiency was not established up to N = 1024. C3 coverage stability failed, and C6 showed substantial cross-seed coordinate instability. ILL_B01_ and ILL_B02_ are therefore fixed-seed illustrative boundary points, not uniquely determined or statistically converged solutions.

Fifth, no older adults, caregivers, clinicians, or design experts evaluated the variables, proxy, renderings, or CAD models. The study provides no evidence concerning perceived safety, comfort, trust, usability, accessibility, or acceptance. Direct evaluation is required, and human–robot interaction, deceleration response, and trust literature identify relevant future concerns [12,13,14,15,20,21,39].

Sixth, no physical prototype was fabricated. The AI-assisted images and Rhino models are illustrative and do not establish structural integrity, manufacturing feasibility, material performance, internal packaging, or cost.

Seventh, representative selection was sensitive to distance definition (C7 overlap = 0.53). The equal-variable distance gives three-quarters of its variable-level contribution to the kinematic block, whereas the block-balanced alternative increases the relative influence of morphology. Neither weighting is empirically validated.

Eighth, the public product benchmark was limited to specifications that could be accessed and authenticated. Product maximum speed was used only as an exploratory anchor and must not be interpreted as an elderly-appropriate approach speed. Population-specific anthropometric standards and older-adult data should be used during later interface validation rather than converted into unverified reach or visibility scores [40,41,42,43,44,45].

Ninth, the submitted Supplementary Materials support summary-level methodological transparency but not independent computational reproduction. Supplementary Table S1 documents the diagnostic definitions, thresholds, aggregation rules, failure actions, hard stops, and observed outcomes, but the Supplementary Materials do not include source code, raw sampled configurations, intermediate diagnostic outputs, implementation records, complete AI prompts, or CAD source files. Independent reproduction from the submitted files alone is therefore not claimed.

### 5.6. Future Work

The next methodological step is to measure system latency, commanded and achieved deceleration, friction-dependent stopping distance, and sensor-trigger behavior on a physical mobile platform. These data would permit replacement of the present idealized constraint with a calibrated stopping model.

Physical prototypes should then be evaluated for shell contact geometry, compliant-material thickness and hardness, support polygon, mass distribution, turning envelope, and emergency stop accessibility. CAD mass-property analysis and dynamic simulation can precede controlled physical tests.

Human-centered studies with appropriate ethics approval should evaluate perceived safety, legibility, trust, comfort, and usability with older adults and caregivers [12,13,14,15]. These studies are also necessary to establish meaningful interface, signaling, and approach behavior parameters.

Finally, Rhino/Grasshopper can be linked directly to the constrained exploration so that selected computational coordinates are transferred without undocumented adjustment. Future visualizations should record the computational coordinate, CAD implementation value, deviation, and reason for deviation.

## 6. Conclusions

This study revised a bio-inspired elderly-care robot workflow into an evidence-tiered, constraint-based, four-dimensional design space exploration. The framework retains protective morphology and pre-contact sensing as delimited functional analogs, while affiliative signaling and homeostatic service logic remain qualitative analogies. Ordinary stability, ergonomic, safety interface, material, and manufacturing decisions are kept outside the biomimetic computation.

The P0 pre-check confirmed that the study-defined domain was feasible for exploration. Across the adaptive N sequence, the I5 distribution became stable, but full coverage and exact representative coordinates did not. No natural clusters were detected, representative selection was distance-sensitive, and N_sufficient_ was not established up to 1024.

ILL_B01_ and ILL_B02_ are therefore used only as fixed-seed illustrative boundary configurations. The AI-assisted visualizations and Rhino models support downstream product design communication, but they are not direct realizations of statistically stable coordinates and were not physically validated.

The main contribution is a transparent workflow that can exclude infeasible combinations, document uncertainty, and support traceable concept translation without claiming an optimal or validated robot. The negative convergence results define the current evidentiary boundary and identify the measurements required before safety, usability, or manufacturing claims can be made.

Future work should calibrate the kinematic model with measured latency and stopping behavior, develop physical prototypes, complete ergonomic and human-subject evaluation, and integrate computational coordinates with CAD through an auditable parameter-transfer process.

## Figures and Tables

**Table 2:** Candidate engineering variables, final computational status, and downstream role.

ID	Variable Name	Unit	Mechanism/Role	Final Range	Final Status	Evidence or Rationale
V01	body height	mm	Product scale	Not screened	Post-screening	Set during CAD and component packaging
V02	body width	mm	Product footprint	Not screened	Post-screening	Set during CAD and indoor-clearance review
V03	body depth	mm	Product footprint	Not screened	Post-screening	Set during CAD and turning-envelope review
V04	base width	mm	Engineering stability	Not screened	Post-screening	Base-width benchmark data were not consistently identifiable
V05	COG height	mm	Engineering verification	Not screened	Post-layout	Requires component mass and placement data
V06	nominal external-shell-edge radius	mm	M1 morphology	10–80	Computational	Author-defined exploratory morphological range
V07	bumper thickness	mm	M1 implementation	Not screened	Post-screening	Material and thickness specification required
V08	compliant-contact implementation strategy	—	M1 implementation	Not screened	Qualitative downstream	Previous continuous coverage ratio was undefined and removed
V09	longitudinal pre-braking trigger distance	m	M2 kinematics	0.5–1.5	Computational	Author-defined exploratory bounds
V10	emergency stop height	mm	Safety interface	Not screened	Post-screening	Standard-constrained interface decision
V11	pre-braking translational speed	m/s	M2 kinematics	0.2–1.0	Computational	Lower bound author-defined; upper bound product-maximum anchored
V12	constant commanded deceleration magnitude	m/s^2^	M2 kinematics	0.2–0.8	Computational	Author-defined exploratory range
V13	visual display center height	mm	M3 interface	Not screened	Post-screening	Requires authenticated anthropometry and viewing geometry
V14	interface angle	°	M3 interface	Not screened	Post-screening	Ergonomic orientation decision
V15	feedback-light height	mm	M3 signaling	Not screened	Post-screening	Visibility model lacked calibrated evidence

**Table 6:** Final D2 diagnostic summary and reporting consequence.

Diagnostic	Definition	Result	Threshold	Status	Interpretation	Reporting Consequence
P0	Feasible acceptance and I5 information	AR = 0.8621; seed-level SD = 0.0016; spread = 0.8177	AR ≥ 0.10; seed-level SD ≤ 0.03; spread ≥ 0.05	PASS	Domain suitable for formal exploration	Full D2 eligible
C1	Paired I5 descriptive statistics	30/30 seeds at 768→1024	≥27/30 seeds	PASS	I5 summaries stable	Distributional stability supported
C2	Paired I5 empirical CDF (KS)	30/30; median D = 0.0249	≥27/30; median ≤ 0.075	PASS	I5 distribution stable	Distributional stability supported
C3	CFRS coverage and variable quantiles	10/30 seeds at 768→1024	≥27/30 seeds	FAIL	Full 4D coverage stability not established	No N_sufficient claim
C4	Cluster tendency gate	Hopkins = 0.53; gap = 0.40; silhouette = 0.18	All gate conditions	N/A	No natural clusters detected	No cluster-derived design types
C6	Cross-seed maximin-set stability	Median matched distance = 0.4515	Full coordinate- and coverage-stability thresholds are reported in Supplementary Table S1.	FAIL	Exact coordinates seed-sensitive	Illustrative points only; H5 triggered
C7	Distance definition robustness	Overlap = 0.53	≥0.75	SENSITIVE	Selection depends on distance weighting	Sensitivity disclosed
Overall	Mandatory diagnostic synthesis	Partial convergence	C1 + C2 + C3 + C6	PARTIAL	I5 stable; coverage/coordinates unstable	Results frozen with explicit limitations

## Data Availability

The original contributions presented in this study are included in the article/Supplementary Material. Further inquiries can be directed to the corresponding author.

## Supplementary Materials

The following supporting information can be downloaded at https://www.mdpi.com/article/10.3390/biomimetics11080578/s1: Supplementary Table S1, “Pre-Specified Diagnostic Definitions, Thresholds, Seed-Aggregation Rules, Failure Actions, and Observed Outcomes for P0 and C1–C7.”. Supplementary Table S1 is provided as the sole Supplementary File for this article. It reports the pre-specified definitions, thresholds, seed-aggregation rules, failure actions, hard stops, and summary outcomes for P0 and C1–C7. Source code, raw sampled configurations, intermediate diagnostic outputs, implementation-correction records, complete AI prompts, and Rhino or Grasshopper source files are not included in the Supplementary Materials. The numerical findings reported in this article should therefore be interpreted as summary-level computational outputs documented in the manuscript and Supplementary Table S1. No human-participant, clinical, or experimental dataset was generated.
